# Novel lamin B receptor mutation (c.561C > G) in a patient with Pelger-Huët anomaly: a case report

**DOI:** 10.3389/fped.2025.1587175

**Published:** 2025-09-04

**Authors:** Jiaojiao Yin, Dan Huang, Zhenya Liu, Enpeng Zhu, Chong Zhang, Linyan Wang, Bing Li

**Affiliations:** ^1^ Department of Clinical Laboratory, Gansu Provincial Maternity and Child-care Hospital, Lanzhou, Gansu, China; ^2^ School of Public Health, Gansu University of Chinese Medicine, Lanzhou, Gansu, China; ^3^ Department of Clinical Laboratory, 940th Hospital of Chinese People’s Liberation Army Joint Support Force, Lanzhou, Gansu, China

**Keywords:** Pelger-Huët anomaly, congenital scoliosis, hemivertebrae, lamin B receptor, nonsense mutation

## Abstract

Pelger-Huët anomaly (PHA), an autosomal dominant disorder characterized by abnormal granulocyte morphology, was first described in 1928. Mutations in the lamin B receptor (*LBR*) gene cause a phenotypic spectrum ranging from isolated PHA, PHA with mild skeletal abnormalities, to the embryonic-lethal Greenberg skeletal dysplasia. We report a Chinese boy presenting peripheral blood granulocyte abnormalities associated with a novel *LBR* gene mutation. Whole-exome sequencing uncovered the *LBR* gene heterozygous mutation, NM_194442.2: c.561C > G (p.Tyr187*). Notably, the patient exhibited scoliosis secondary to hemivertebrae, potentially representing a previously unreported skeletal manifestation of mutations in the *LBR* gene. Analyzing the differential diagnosis between PHA, immature granulocytes, and pseudo-PHA, along with elucidating genotype-phenotype correlations for *LBR* mutations, is crucial for advancing our understanding of PHA and related disorders.

## 1 Introduction

Pelger-Huët anomaly (PHA) is an inherited morphologic disorder characterized by granulocyte nuclear hyposegmentation, typically manifesting as dumbbell-shaped or spectacle-like bilobed nuclei with coarse chromatin clumping (1). This autosomal dominant condition has been molecularly linked to chromosome 1q41-43 (2), specifically attributed to pathogenic variants in the lamin B receptor (*LBR*, MIM 600024) gene at 1q42.1 (3). While PHA is classically considered a benign hematologic finding, emerging evidence suggests potential associations with mild skeletal dysplasias (4). Herein, we report a pediatric case of genetically confirmed PHA exhibiting severe congenital scoliosis secondary to hemivertebrae formation, coupled with a novel heterozygous missense mutation in the *LBR* gene (c.561C > G).

## 2 Case report

**Figure 1:**
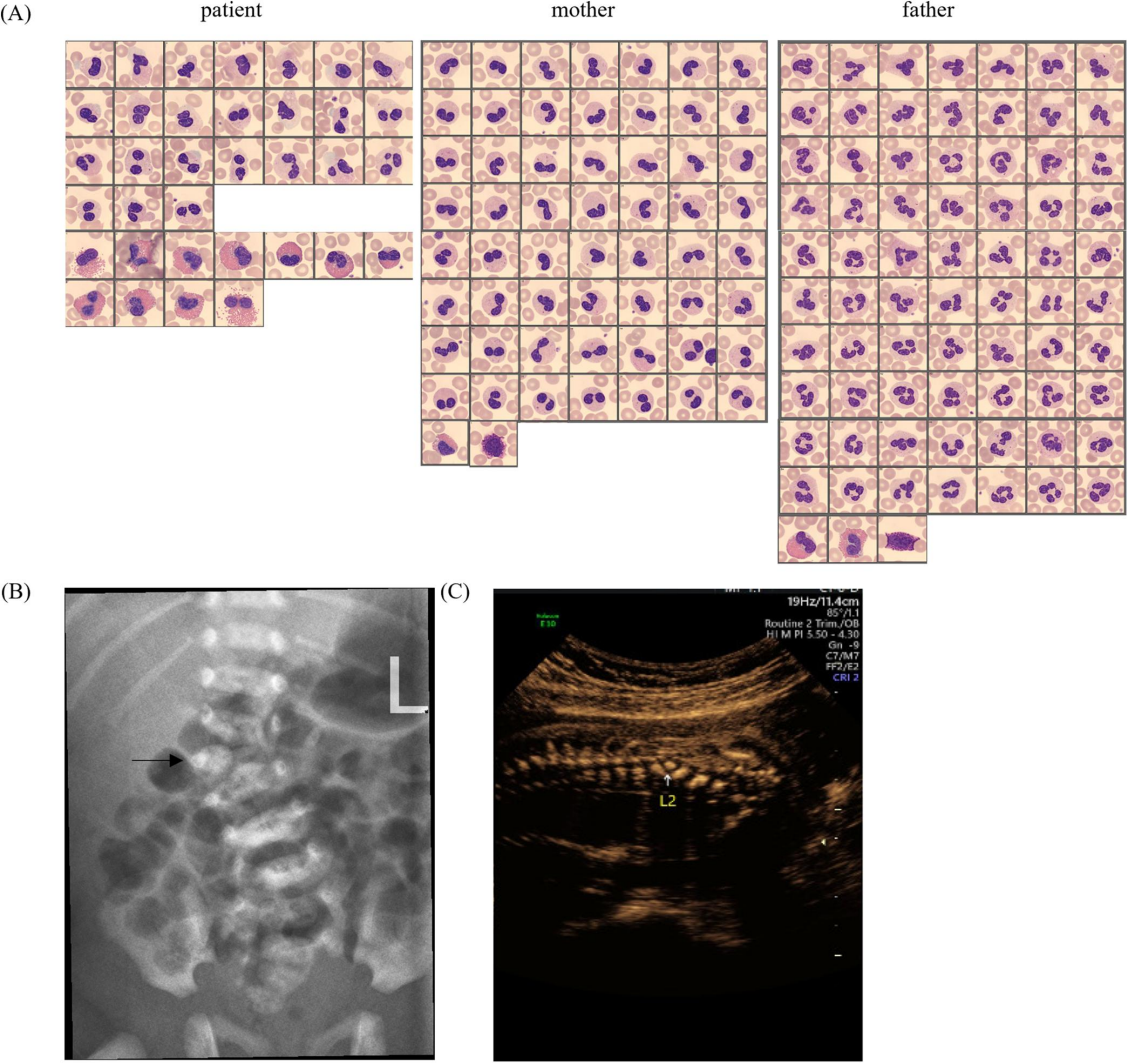
**(A)** Peripheral blood smear micrographs from the patient, mother, and father, showing Giemsa-stained granulocytes. **(B)** Lateral spinal radiograph (X-ray) with an arrow indicating a specific vertebral anomaly. **(C)** Prenatal ultrasound image focusing on the L2 vertebra, demonstrating abnormal ossification.

A 2-year-old boy was admitted to our hospital for physical examination. Routine peripheral blood examination showed the white blood cell count of 6.13 × 10^9^/L with normal differential counts (neutrophils 29.2%, eosinophils 8.4%, basophils 0.8%, monocytes 8.4%, and lymphocytes 53.2%). However, the percentage of immature granulocytes (IG%) is 2.4% that violated the routine blood retest rules and the Sysmex DI-60 analyzer was rechecked with an automatic pusher. A total of 110 cells were randomly analyzed including 17 neutrophils (5 mono-lobed and 12 bi-lobed nuclei) and 11 eosinophils (6 mono-lobed and 5 bi-lobed nuclei) (Figure 1A). To rule out hereditary PHA, we contacted the boy's biological parents for a peripheral blood smear and confirmed the existence of similar neutrophil-related morphological abnormalities in his biological mother while the boy's biological father had normal granulocytes (Figure 1A). Based on the physical examination and medical history, the boy was found to have scoliosis. Spinal radiographs showed that he had lumbar scoliosis and lumbar 2-vertebral hemivertebral deformity (Figure 1B). His mother was found fetal scoliosis at 23 weeks of pregnancy by systematic color Doppler ultrasound screening (Figure 1C).

From birth to present, the child had a regular health check-up almost every three months. Clinical features assessed showed slightly lower head circumference, length, and weight when plotted on a standard WHO chart (Supplementary Figures S1A–C). The serum thyroid-stimulating hormone and genetic metabolites levels are within the normal ranges (Supplementary Table S1). The levels of 25-hydroxyvitamin D, serum ferritin and trace elements (including calcium, iron, zinc, lead, copper, cadmium, potassium, sodium, magnesium) were almost within the normal ranges (Supplementary Table S2). At postnatal 9 and 18 months, the Gesell Developmental Schedules (GDS) score (5) were under 86 and more than 85, respectively (Supplementary Figure S1D). Overall, the boy had slightly stunted growth but normal mental development.

**Figure 2:**
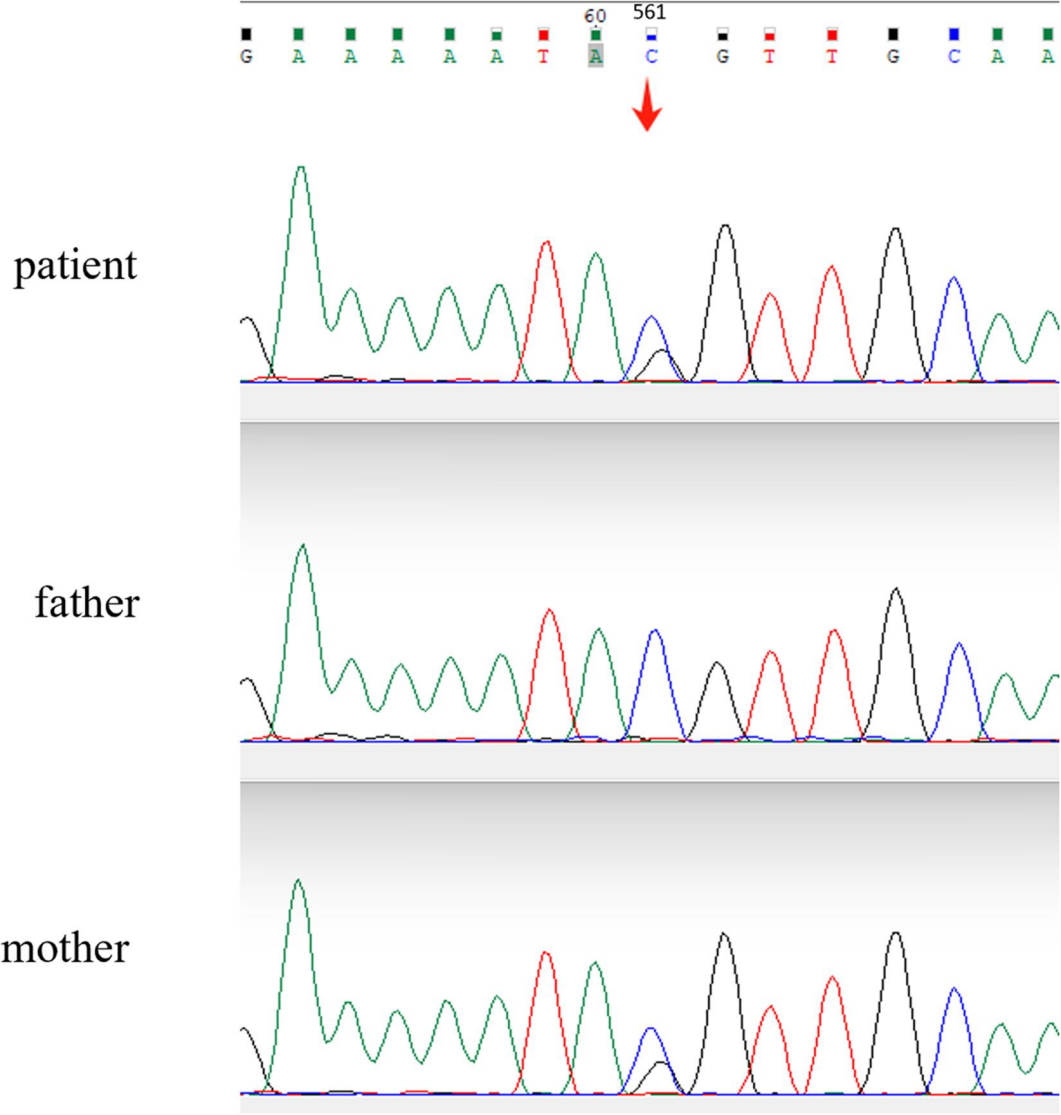
Sanger sequencing chromatograms comparing the LBR gene sequences of the patient and parents. A heterozygous mutation is evident in the patient's sequence (arrow), where a nucleotide substitution from cytosine (C) to guanine (G) occurs at position c.561, resulting in a missense variant.

To identify the potential gene mutation, the Whole-exome high-throughput sequencing is used, including exons of approximately 20,000 genes in the human genome and the mitochondrial genome. A new mutation site in the *LBR* gene was discovered: c.561C > G (p. Tyr187*). Sanger sequencing of the boy and his parents validated the candidate *LBR* gene variant that both the child and his mother were found to have heterozygous mutations at the locus (Figure 2). According to the ACMG (American College of Medical Genetics and Genomics) guidelines, this variant has been preliminarily classified as pathogenic: PVS1 + PP4 + PM2.

## 3 Discussion

PHA was first discovered by Pelger in the peripheral blood of two tuberculosis patients, mistakenly believing that the nuclear was shifted to the left due to infection (6). The IG fraction includes promyelocytes, myelocytes and metamyelocytes (blasts and band cells are not included). Elevated IG% is common in the peripheral blood of patients with bacterial infections, neonates, pregnant women, and patients taking recombinant human granulocyte colony-stimulating factor(rhG-CSF) (7–9). When IG% is elevated, mild- to late myelocytes are seen on peripheral blood smear but not in PHA. Secondly, the large amount of chromatin condensation observed in the nucleus of PHA granulocytes can help distinguish PHA from the “left shift” of neutrophils. The Figure 1A shows the neutrophils of the child and his mother have a large amount of chromatin condensation compared to the father.

PHA needs to be differentiated from pseudo-PHA which is an acquired neutrophil dysplasia similar to PHA. This abnormality is frequently associated with myelodysplastic syndromes and also described in some clinical situations, especially under the effect of certain drugs (10). Congenital PHA is associated with 55% to 95% of circulating neutrophils exhibiting classic dysmorphology (11) and the frequency of pseudo-PHA neutrophils has ranged between 77% and 99% (12). We cannot distinguish PHA from pseudo-PHA by abnormal granulocyte ratios, however, pseudo-PHA has morphologically normal eosinophils and basophils (12), which are abnormal in PHA (Figure 1A). Additionally, differentiation can be achieved by taking a detailed patient medical history and performing morphological analysis of neutrophils in first-degree relatives.

The *LBR* gene contains 35 kb bases, encodes 615 amino acids, and has 13 coding regions, of which 1–4 are N-terminus, nucleoplasmic domain, and 5–13 are C-terminus, hydrophobic domain. Two seemingly unrelated functions have been attributed to *LBR*. The nucleoplasmic domain is associated with lamin B, chromatin, and other proteins causing crucial changes in nuclear architecture (3), while its transmembrane domains exhibit sterol reductase activity (13). Deletion of *LBR* N-terminal domains using CRISPR/Cas-9 gene editing technology in the mouse affected the morphology and chromatin organization of white blood cells but not skin or skeletal defects (14). However, in human there is no evidence that different phenotypes are associated with different *LBR* domains (Table 1). Genetic variation in *LBR* affects the expression of *LBR* protein, which in turn correlates with a continuum of clinical manifestations ranging from no phenotype to isolated PHA through PHA with mild skeletal dysplasia to Greenberg skeletal dysplasia (Table 1). Mutations in the *LBR* gene is a form of nuclear envelopathies and exhibit the characteristic phenotype of causing PHA (15). So far, we have found point mutation, splice site, frameshift and nonsense mutations in *LBR* (16). Heterozygous *LBR* mutations lead to nuclear hyposegmentation of neutrophils without causing disease, except for missense mutations such as p. Arg583Gln which did not alter nuclear shape in neutrophils (17). However, patients with homozygous/compound heterozygous mutations in *LBR* can lead to severe perinatal fatal autosomal recessive skeletal dysplasia, Greenberg skeletal dysplasia, and even those who survive are accompanied by severe skeletal dysplasia (Table 1). In this respect, heterozygote for mutation p. Tyr187* was associated with PHA with scoliosis. Notably, we also found that nonsense mutations, base pair insertions or deletions causing frame shifts that create premature stop codons can all lead to PHA (Table 1).

**Table 1:** Congenital disorders associated with mutations in the lamin B receptor gene.

Mutation type	Mutation (gene)	Mutation (protein)	Class of mutation	Variants (exons)	Pelger-Huet anomaly	Clinical features	Ref.
Homozygous	c.1366C > G	p. Leu456Val	Point mutation	11	N/A	Non-lethal skeletal dysplasia; scoliosis without hemivertebrae	(21)
	c.1534C > T	p. Arg512Trp	Point mutation	12	N/A	Non-lethal skeletal dysplasia; scoliosis without hemivertebrae	(17)
	c.1748G > A	p. Arg583Gln	Point mutation	13	Unknown	Greenberg Dysplasia	(22)
	c.1757G > A	p. Arg586His	Point mutation	13	Unknown	Greenberg Dysplasia	(23)
	c.1492delT	p. Tyr468Thrfs475*	Frameshift	12	Unknown	Greenberg Dysplasia	(16)
	c.1639A > G	p. Asn547Asp	Point mutation	13	Unknown	Greenberg Dysplasia	(24)
	c.1599_1605TCTTCTAdelinsCTAGAAG	p.534*	Frameshift	12	Unknown	Greenberg Dysplasia	(25, 26)
Compound heterozygous	c.32delTGGT/c.1748G > A	p.Val11Glufs24*/p. Arg583Gln	Frameshift/point mutation	1/13	Unknown	Greenberg Dysplasia	(16)
	c.1757G > A/c.1687 + 1G > A	p. Arg586His/	Point mutation/splice donor	13/13	Unknown	Greenberg Dysplasia	(27)
	c.1757G > A/c.194delG	p. Arg586His/p. Gly65Valfs53*	Point mutation/nonsense	2/13	Unknown	Greenberg Dysplasia	(28)
	c.1504C > G/c.1748G/T	p. Arg502Gly/p. Arg583Lu	Point mutation	12/13	Yes	Non-lethal skeletal dysplasia Platyspondyly	(17)
	c.1757G > C/c.43C > T	p. Arg586Ser/p. Arg15*	Point mutation/nonsense	1/14	Yes	Non-lethal skeletal dysplasia	(17)
	c.651_653delinsTGATGAGAAA/c.1757G > A	p. Ile218Aspfs19*/p. Arg586His	Frameshift/point mutation	6/14	Yes	Non-lethal skeletal dysplasia	(4)
	c.226C > T/c.1640A > G	p. Arg76*/p. Asn547Ser	Nonsense/point mutation	3/13	Yes	Non-lethal skeletal dysplasia Platyspondyly	(29)
	c.1174G > A/c.1535G/A	p. Gly392Arg/p. Arg512Gln	Point mutation	9/12	N/A	Non-lethal skeletal dysplasia	(30)
Heterozygous	c.43C > T	p. (Arg15*)	Nonsense	1	Yes	Skeletal abnormalities (such as osteochondroma), cognitive impairment, and hearing loss (DFNB4/EVA mutation).	(31)
	c.1129C > T	p. Arg377*	Nonsense	9	Yes	unknown	(3)
	c.1308G > A	p. Trp436*	Nonsense	10	Yes	unknown	(3)
	**c.561C** **>** **G**	p. Tyr187*	**Nonsense**	**4**	**Yes**	**scoliosis with hemivertebrae**	**New**
	c.32delTGGT	p. Val11Glufs24*	Nonsense	1	Yes	No severe skeletal or metabolic abnormalities.	(16)
	c.500G > C;501-504delCCTT	p. Ser167Thrfs176*	Frameshift	4	Yes	Unknown	(3)
	c.1173del	p. Gly392Aspfs393*	Frameshift	9	Yes	Unknown	(3)
	c.1599_1605TCTTCTAdelinsCTAGAAG	p.534*	Frameshift	12	Yes	Health	(25, 26)
	c.1748G > A	p. Arg583Gln	Point mutation	13	N/A	Health	(16)
	c.355C > T	p. Pro119Leu	Point mutation	3	Yes	Health	(32)
	c.1706C > G	p. Pro569Arg	Point mutation	13	Yes	Health	(32)

Bold type represents the novel LBR gene variant sites identified in this study.

In previous reports, skeletal abnormalities associated with *LBR* gene mutations included short stature, short upper extremities, short metacarpal bones, postaxial polydactyly, and kyphosis, with some cases showing reduced severity and spontaneous resolution of skeletal manifestations and imaging features with age (18). Although scoliosis has been reported in previous cases, scoliosis due to the hemivertebrae has been reported for the first time. In this case, the skeletal abnormality manifests as congenital scoliosis, a form of vertebral malformation with a genetic susceptibility (19). The genetic basis of congenital scoliosis is complex, involving mutations in multiple genes, particularly those related to the Notch signaling pathway, such as *TBX6* and *LFNG* (20). This report provides additional evidence of variability for *LBR*-related disorders associated with Pelger-Huët anomaly, i.e., congenital scoliosis caused by hemivertebrae, which is not spontaneous regression.

## 4 Conclusion

This case report describes a novel *LBR* mutation identified in a child presenting with PHA and hemivertebrae, further expanding the known phenotypic spectrum associated with *LBR* mutations. Our report details a heterozygous mutation. Analysis of current literature on congenital disorders linked to *LBR* gene mutations reveals that individuals with heterozygous *LBR* mutations are generally healthy apart from the characteristic features of PHA. This finding highlights the necessity of early PHA diagnosis, which facilitates preimplantation genetic testing (PGT) implementation to block intergenerational transmission of the pathogenic variant. Whether the occurrence of congenital scoliosis in this child is associated with the *LBR* gene mutation requires additional cases and functional studies to elucidate the underlying mechanisms and refine therapeutic strategies.

## Data Availability

The original contributions presented in the study are included in the article/Supplementary Material, further inquiries can be directed to the corresponding author.
