# Supplementary material for: Novel lamin B receptor mutation (c.561C > G) in a patient with Pelger-Huët anomaly: a case report
Source: Front Pediatr. 2025 Sep 4;13:1587175. doi: 10.3389/fped.2025.1587175 (PMC12443853; doi:10.3389/fped.2025.1587175)
Supplement: Supplementary file 1 [file Supplementaryfile1.docx]

Supplementary Material

# Supplementary Figures and Tables

## Supplementary Figures


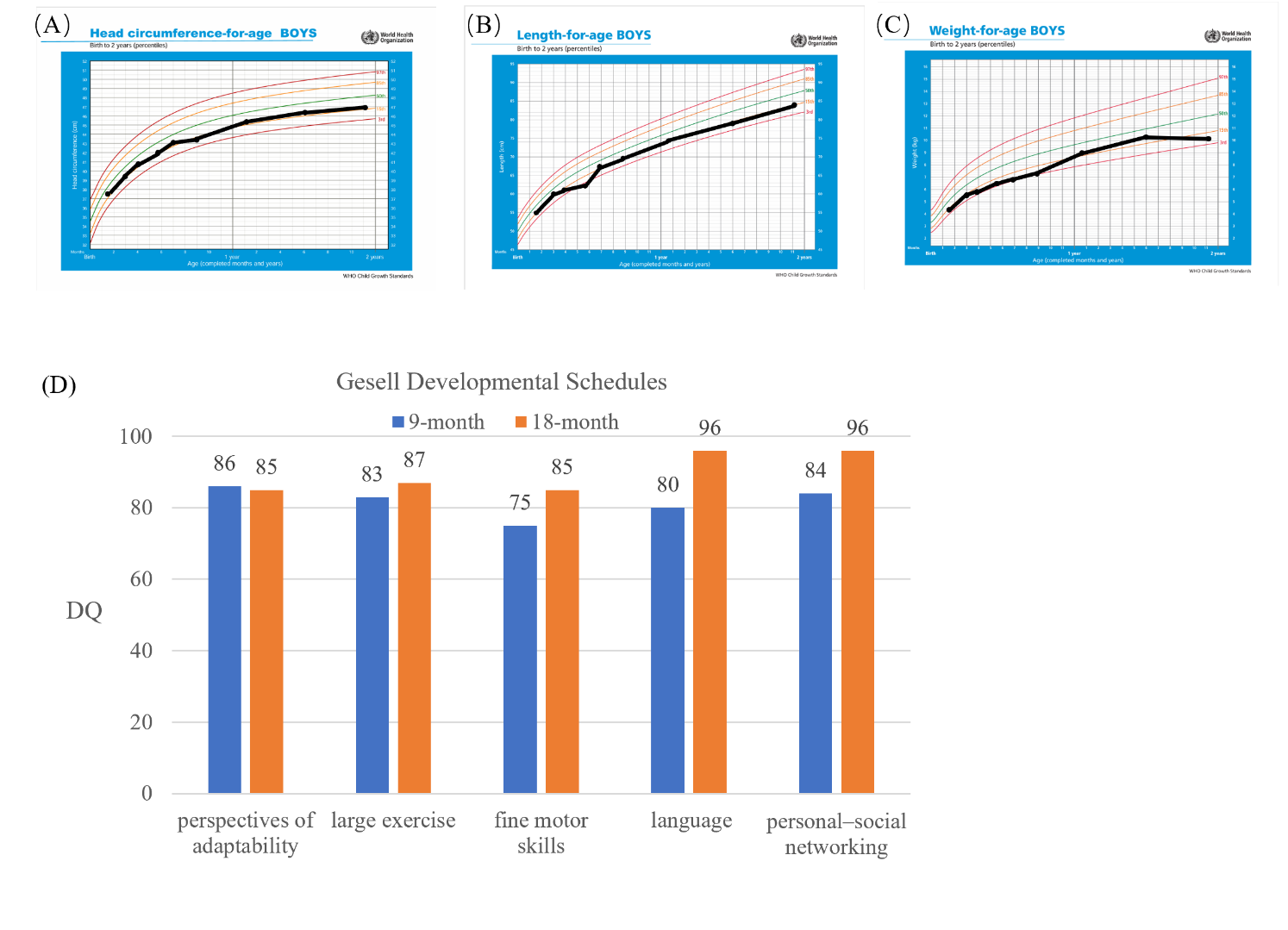


**Supplementary Figure 1.** (A) The boy's birth - 2 years old head circumference-for-age curve;(B) The boy's birth - 2 years old length-for-age curve; (C) The boy's birth - 2 years old weight-for-age curve. (D) The boy's 9-month and 18-month Gesell development schedule.

## Supplementary Tables

Supplementary Table 1 Laboratory evaluation.

|  | 6 Months | 8 Months | 12 Months | 18 Months | 24 Months | Reference range |
| --- | --- | --- | --- | --- | --- | --- |
| 25-hydroxyvitamin D (ng/ml) | 24.75↓ | 45.05 | 34.57 | 35.11 |  | Lacking:<20  Insufficient:20-30  Sufficient:30-100  Poisoned:>100 |
| Peripheral blood ferritin (ng/ml) | 76.56 | 81.92 |  | 89.13 | 123.17 |  |
| calcium (mmol/L) | 1.99 |  | 1.72 | 1.72 |  | 1.64-2.23 |
| iron (mmol/L) | 8.01 |  | 7.88 | 8.33 |  | 7.0-9.0 |
| zinc (μmol/L) | 48.11 |  | 53.31 | 56.67 |  | 50.74-90.36 |
| lead (μg/dL) | 2.86 |  | 4.81 | 3.9 |  | <10 |
| copper (μmol/L) | 18.82 |  | 17.09 | 20.03 |  | 9.61-28.8 |
| cadmium (μg/L) | 1.16 |  | 2.12 | 0.77 |  | <5 |
| potassium (mmol/L) | 36.98 |  | 44.2 | 42.34 |  | 37.09-49.11 |
| sodium (mmol/L) | 87.44 |  | 79.05 | 70.1 |  | 31-98 |
| magnesium (mmol/L) | 1.45 |  | 1.72 | 1.62 |  | 1.24-1.79 |
| Serum thyroid-stimulating hormone（μU/mL） |  |  |  |  | 0.98 | <8 |

Supplementary Table 2 Laboratory evaluation for inherited metabolic disorders.

| Abbreviation of the test item | Result  (μmol/L) | Reference range | Abbreviation of the test item | Result  (μmol/L) | Reference range | Abbreviation of the test item | Result  (μmol/L) | Reference range |
| --- | --- | --- | --- | --- | --- | --- | --- | --- |
| ALA | 493.23 | 172-1053 | C5 | 0.10 | 0.05-0.53 | C18:2 | 0.35 | 0.07-0.66 |
| ARG | 39.01 | 2.0-44.0 | C5:1 | 0.01 | 0.00-0.03 | C3/C2 | 0.11 | 0.05-0.27 |
| CIT | 27.93 | 6-52 | C5DC_C6OH | 0.13 | 0.05-0.41 | C4/C2 | 0.02 | 0.00-0.10 |
| GLY | 658.60 | 190-1255 | C6 | 0.02 | 0.01-0.13 | C5/C2 | 0.01 | 0.00-0.11 |
| LEU | 319.52 | 79-350 | C6DC | 0.14 | 0.03-0.29 | C8/C2 | 0.00 | 0.00-0.03 |
| MET | 32.93 | 6-34 | C8 | 0.02 | 0.01-0.14 | C14:1/C16 | 0.04 | 0.01-0.21 |
| ORN | 222.62 | 28-318 | C8:1 | 0.20 | 0.01-0.52 | C16-OH/C16 | 0.04 | 0.00-0.03 |
| PHE | 96.78 | 24-100 | C10 | 0.02 | 0.02-0.19 | C14:1/C8:1 | 0.04 | 0.13-2.27 |
| PRO | 264.42 | 95-418 | C10:1 | 0.04 | 0.02-0.21 | C0/(C16+C18) | 26.91 | 2.98-48.97 |
| TYR | 140.53 | 40-278 | C10:2 | 0.01 | 0.02-0.21 | C3/Met | 0.04 | 0.03-0.45 |
| VAL | 324.64 | 63-298 | C12 | 0.02 | 0.02-0.22 | C3/C0 | 0.04 | 0.01-0.18 |
| ARG/ORN | 0.18 | 0.02-0.64 | C12:1 | 0.02 | 0.01-0.26 | C5/C0 | 0.00 | 0.00-0.02 |
| CIT/ARG | 0.72 | 0.09-9.86 | C14 | 0.05 | 0.05-0.39 | C5/C3 | 0.08 | 0.03-0.51 |
| MEET/PHE | 0.34 | 0.07-4.11 | C14:1 | 0.03 | 0.02-0.29 | (C3DC_C4OH)/C4 | 0.32 | 0.14-1.59 |
| ORN/CIT | 7.97 | 0.95-16.13 | C14OH | 0.00 | 0-0.03 | (C3DC_C5OH)/C0 | 0.01 | 0.0-0.02 |
| ORN/PHE | 0.69 | 0.18-1.09 | C14:2 | 0.01 | 0.01-0.07 | (C4DC_C5OH)/C8 | 20.50 | 1.39-18.08 |
| LEU/PHE | 5.03 | 0.02-21.9 | C16 | 0.82 | 0.47-6.34 | (C5DC_C6OH)/C8 | 6.50 | 0.83-14.00 |
| GLY/PHE | 2.30 | 0.15-4.83 | C16OH | 0.01 | 0.01-0.05 | C14:1/C12:1 | 1..50 | 0.75-5 |
| C0 | 3.30 | 0.49-6.88 | C16:1 | 0.02 | 0.02-0.39 | C8/C10 | 1..00 | 0.38-2.00 |
| C2 | 6.81 | 1.3-39.3 | C16:1-OH | 0.03 | 0.01-0.13 | C5DC_C6OH/C3 | 0.11 | 0.03-0.48 |
| C3 | 33.91 | 0.3-4.95 | C18 | 0.44 | 0.21-1.68 | C16/C2 | 0.07 | 0.06-1.33 |
| C3DC_C4OH | 11.16 | 0.02-0.36 | C18OH | 0.01 | 0.00-0.03 | C16/C3 | 0.68 | 0.40-5.61 |
| C4 | 1.21 | 0.08-0.51 | C18:1 | 0.82 | 0.39-2.75 |  |  |  |
| C4DC_C5OH | 0.41 | 0.09-0.55 | C18:1-OH | 0.01 | 0.01-0.06 |  |  |  |
